# Steroid hormones dynamics during coral reproduction: Multi-year patterns in *Acropora eurystoma* from the Red Sea

**DOI:** 10.1016/j.isci.2026.116205

**Published:** 2026-06-02

**Authors:** Chen Azulay, Karine Kleinhaus, Maoz Fine

**Affiliations:** 1 Department of Ecology, Evolution and Behavior, The Alexander Silberman Institute of Life Sciences, The Hebrew University of Jerusalem, Edmond J. Safra Campus, Jerusalem 91904, Israel; 2 The Interuniversity Institute for Marine Sciences, POB 469, Eilat 88103, Israel; 3 School of Marine and Atmospheric Sciences, Stony Brook University, Stony Brook, NY 11794-5000, USA

**Keywords:** Ecology, Evolutionary biology, Systems biology

## Abstract

Sex steroid hormones have been identified in coral tissues and associated primarily with spawning events, yet successful spawning relies on the precisely timed gametogenesis. We investigated steroid hormone dynamics during gametogenesis in *Acropora eurystoma* from the Gulf of Aqaba over three reproductive cycles (2021–2023). Estrogen peaked in March, declined toward May, and rose by spawning (June-July). This pattern was inversely correlated with oocyte diameter, suggesting association between estrogen and gametogenic stage. Progesterone remained stable during reproductive months but peaked in October, potentially signaling initiation of the next reproductive cycle. Photoperiod and UV radiation were stronger predictors of estrogen levels than temperature, suggesting light-related cues may influence coral endocrine regulation. Despite uniform estrogen distribution, oocyte prevalence was higher in central than peripheral regions, indicating polyp-level modulation of colony-wide hormonal signals. These findings suggest hormonal profiling as an early-warning system for detecting reproductive disruption, with implications for coral conservation and restoration.

## Highlights

• First multi-year tracking of steroid hormones across coral gametogenesis
• Estrogen peaks in early vitellogenesis (March), declines as oocytes mature
• Photoperiod and UV radiation are stronger predictors of estrogen than temperature
• Oocyte prevalence 5.7-fold higher in central vs. peripheral colony regions

## Graphical abstract

**Figure:**
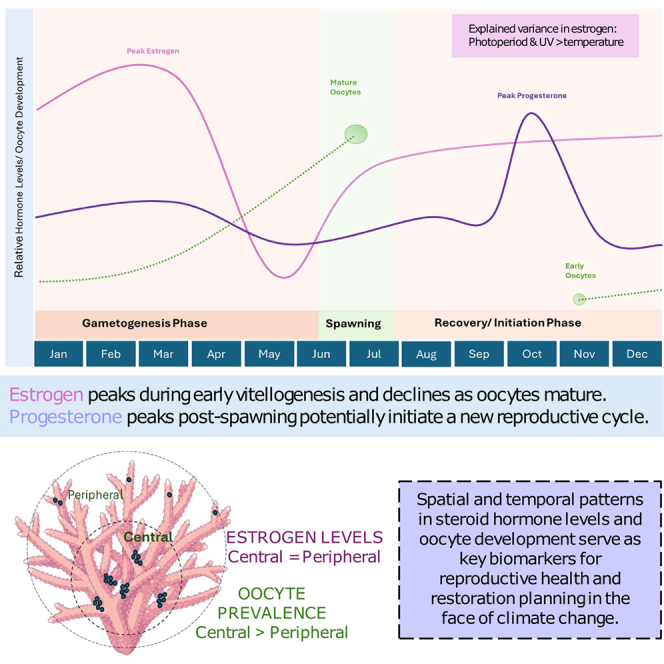

## Introduction

Rising ocean temperatures are disrupting many marine ecosystems, with coral reefs among the most affected.^1^ Coral reef recovery depends on successful reproduction, yet these cycles are increasingly vulnerable to environmental change.^2^^,^^3^

Most reef-building corals are hermaphroditic broadcast spawners.^4^^,^^5^ Spawning events are highly synchronized to specific months, nights, and hours, and are regulated by environmental cues, primarily water temperature and lunar phase.^6^^,^^7^^,^^8^^,^^9^ This synchrony must extend beyond gamete release to include gametogenesis itself, ensuring gametes reach maturity in time for spawning. Oogenesis typically requires an average of six months or more, while spermatogenesis generally begins a couple of months before gamete release into the water column.^4^^,^^10^ Yet the regulatory mechanisms coordinating this months-long process remain ambiguous.

In vertebrates gametogenesis is regulated by steroid hormones.^11^^,^^12^ Vertebrate-type steroids have been identified across marine invertebrates, where they correlate with gametogenic progression and oocyte development, suggesting a conserved role in reproductive regulation.^13^^,^^14^^,^^15^ Sex steroid hormones such as estrogen (estrone [E1], 17β-estradiol [E2], and estriol [E3]), progesterone, and testosterone have also been identified in several reef-building coral species.^16^^,^^17^ Steroidogenic enzymes including aromatase, the key enzyme for estrogen synthesis, have been detected in corals,^17^^,^^18^ suggesting endogenous production. Early studies detected peak E2 levels in seawater during spawning events^19^ and in coral tissues one day prior to spawning,^20^ suggesting endogenous production coinciding with gamete release. More recently, Tan et al.^21^ detected E2 several months before spawning, during oocyte development stages (vitellogenic months). E2 was absent post-spawning, suggesting estrogen corresponds to oocyte nutrient accumulation rather than final spawning preparation. Additionally, experimental exposure to elevated estrogen levels has been shown to reduce spawning output.^22^ While the functional role of these hormones in corals remains unclear, their temporal association with reproductive events suggests potential involvement in regulating gametogenesis timing and oocyte development.

Progesterone has been detected in the soft coral *Sinularia polydactyla*, with elevated levels in the months following spawning,^14^ while relatively stable levels were found during the gametogenesis period in reef-building corals *Pocillopora damicornis*^18^ and *Acropora tenuis*.^21^ However, comprehensive temporal patterns across complete annual reproductive cycles, spatial hormone distribution within colonies, and environmental drivers during gametogenesis remain largely uncharacterized.

Beyond the temporal complexity of coral reproduction, reproductive effort is not uniformly distributed within individual coral colonies. Studies across various coral species have documented spatial heterogeneity in reproductive effort, with central parts of colonies consistently showing greater fertility and fecundity compared to peripheral areas.^23^^,^^24^ In branching corals like *Acropora* species, central regions often harbor older, more developed polyps compared to actively growing peripheral areas,^24^^,^^25^ suggesting that polyp age and developmental stage may influence reproductive capacity. Investigating whether steroid hormones vary spatially within a colony, or whether uniform hormonal signals translate to heterogeneous reproductive responses, could shed light on the local factors that modulate reproductive effort at the polyp level.

We characterized estrogen and progesterone levels across three consecutive reproductive cycles in *Acropora eurystoma* in the Gulf of Aqaba (GoA), addressing three unresolved questions. First, do hormone levels correlate with gametogenic progression throughout the annual cycle,^21^ or only peak before spawning as previously reported?^17^^,^^19^^,^^20^ Second, do hormone levels vary spatially within colonies, potentially explaining reproductive heterogeneity?^23^^,^^24^ Third, which environmental factors correlate with hormone dynamics during gametogenesis?

*A. eurystoma* is a hermaphroditic broadcast spawner. In the GoA, the gametogenic cycle has been recorded from January and spawning typically occurs in June-July.^26^ This months-long cycle provides an ideal model for investigating hormonal regulation throughout the complete reproductive period. Moreover, corals in the GoA exhibit exceptional heat tolerance, establishing this region as an important thermal refuge.^27^^,^^28^^,^^29^^,^^30^^,^^31^ Characterizing baseline endocrine dynamics across complete reproductive cycles in these resilient populations provides a critical reference for understanding coral reproductive physiology and informing conservation strategies in a changing ocean.

## Results

### Temporal patterns in hormone levels

#### Chemical analysis of seawater *in situ*

Seawater E2 concentration was mostly below the detection limit (<0.058 ng/L), with only 4 out of 9 C–18 disc sampler groups showing detectable levels (0.10–2.53 ng/L), confirming our study site as suitable for investigating endogenous hormone dynamics (Table S3).

#### Steroid hormone determination in coral tissue

**Figure 1:**
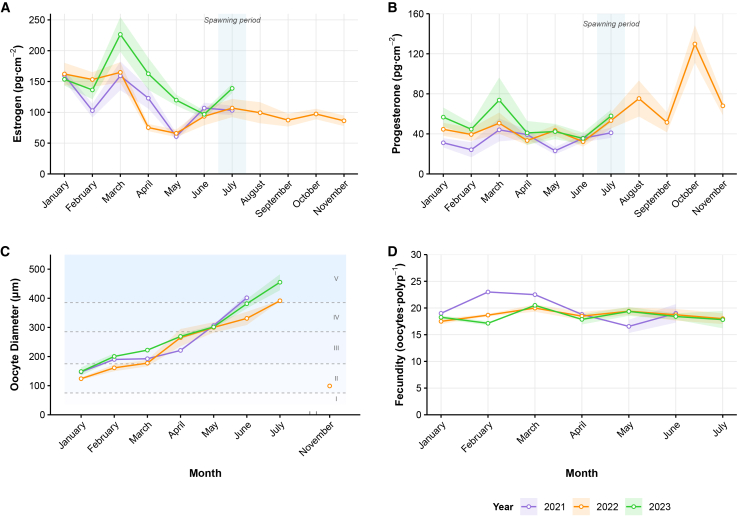
Seasonal variation in steroid hormones and reproductive traits in *Acropora eurystoma* (2021–2023) (A) Estrogen peaked in March, declined through May and rose again toward spawning (GAMs: 26%–44% deviance explained, *p* < 0.001). (B) Progesterone remained stable during the reproductive season (January-July) but showed a marked October increase in 2022 (204% higher than reproductive season, *p* < 0.001). (C) Oocyte diameter followed a sigmoidal growth pattern to July (GAM: 87.6% deviance, *p* < 0.001); Developmental stages (I-V) adapted from Tan et al. (2020): stage I (35–75 μm), stage II (76–175 μm), stage III (176–285 μm), stage IV (286–385 μm), stage V (>386 μm). (D) Fecundity was stable across months (mean 18.6 ± 0.2 oocytes·polyp^−1^). Lines show monthly means by year (purple: 2021, orange: 2022, green: 2023, shaded areas: ±SE). Light blue shading in A and B indicates the typical spawning period (mid-June to mid-July).

Estrogen exhibited clear seasonal patterns across all three reproductive cycles (Figure 1A). Concentrations peaked in March and declined toward May, then recovered by the spawning period (June-July). GAMs explained up to 43.9% (2021–2022) and 26.3% (2023) of temporal variance (Table 1, detailed outputs in Table S5, all *p* < 0.001). Estrogen levels in January-March exceeded those in May-June across all years (Kruskal-Wallis with post-hoc Dunn’s tests, *p* < 0.05, Table S6). March peak estrogen levels varied in magnitude between years (2021: 159.2 pg/cm^2^; 2022: 164.8 pg/cm^2^; 2023: 226.4 pg/cm^2^), yet the temporal pattern was reproducible across all three consecutive reproductive cycles (Table 1).

**Table 1:** Statistical significance and deviance explained (%) of temporal patterns in steroid hormones concentrations and reproductive parameters analyzed by generalized additive models

Parameter	2021	2022	2023	Combined Model
Estrogen	significant (39.6%)	significant (43.9%)	significant (26.3%)	significant (24.2%) *n* = 247
Progesterone	NS (<1%)	NS (<10%)	NS (<1%)	significant (17.7%) *n* = 263
Oocyte Diameter	significant (90.4%)	significant (84.6%)	significant (88.1%)	significant (87.6%) *n* = 86
Fecundity	NS (20.2%)	NS (<1%)	NS (<1%)	NS (3.9%) *n* = 83

Individual years include only reproductive months (2021–2023); while the combined model includes both reproductive and non-reproductive months. Significant (*p* < 0.05); NS = Not significant. Complete GAM statistics are provided in Table S5.

Progesterone levels remained stable during reproductive months (January-July; Figure 1B), with no clear seasonal pattern, as evidenced by GAM models explaining <1% of variance in individual years (Table 1; detailed outputs in Table S5). However, when 2022 sampling was extended through non-reproductive months (August-November), an October peak was observed (129.8 ± 18.6 pg/cm^2^), 204% higher than reproductive season values (*p* < 0.001), with the annual model explaining 17.7% of variance (Table 1).

#### Gametogenesis progress and fecundity

Oocyte diameter followed sigmoidal growth from January through July (Figure 1C), with combined GAM explaining 87.6% of variance (Table 1, *n* = 86). Gametogenesis progressed from stage II (immature, January-February, 120–200 μm) through stage III (early vitellogenic, February-May, 177–269 μm) and stage IV (late vitellogenic, May-June, 300–381 μm) to stage V (matured, June-July, 391–455 μm). Oocytes exhibited slow initial growth (January-March), followed by accelerated development and final maturation (Table 2). Oocytes were absent during the non-reproductive period (August-October); stage I oocytes were detected in November 2022, indicating initiation of the next reproductive cycle.

**Table 2:** Monthly progression of oocyte diameter and growth rates across three reproductive seasons (2021–2023)

Year	Month	Mean Diameter (μm)	Growth Rate (μm/month)	*n*	Total Colonies	% with Oocytes
2021	January	147.0	–	1	11	9
	February	190.0	43.0	1	12	8
	March	192.5 ± 27.5	2.5	2	11	18
	April	221.0	28.5	1	12	8
	May	306.3 ± 3.5	85.3	3	12	25
	June	401.7 ± 11.9	95.3	3	12	25
2022	January	124.0 ± 4.0	–	2	11	18
	February	161.3 ± 11.6	37.3	4	11	36
	March	177.3 ± 11.2	16.0	6	11	55
	April	265.0 ± 29.6	87.7	4	11	36
	May	300.0 ± 18.1	35.0	5	10	50
	June	331.0 ± 22.9	31.0	5	9	56
	July	391.5 ± 1.5	60.5	2	9	22
2023	January	148.5 ± 14.4	–	3	8	38
	February	200.4 ± 10.4	51.9	11	8	88
	March	222.0	21.6	1	8	13
	April	269.0 ± 9.0	47.0	4	8	50
	May	301.7 ± 6.1	32.7	10	9	67
	June	381.5 ± 16.2	79.8	13	9	78
	July	455.2 ± 27.4	73.6	5	9	44

*n* = number of fragments with detectable oocytes (>100 μm) across all sampling points within that month. Each fragment represents 5 dissected polyps. Total Colonies = number of colonies sampled that month. Growth rate calculated as difference in mean diameter between consecutive months within each year. Values are mean ± SE where *n* ≥ 2.

Fecundity remained stable across the reproductive season (18.6 ± 0.2 oocytes·polyp^−1^; Figure 1D), with GAM models explaining <4% of temporal variance and showing no significant trends in any year (Table 1).

**Figure 2:**
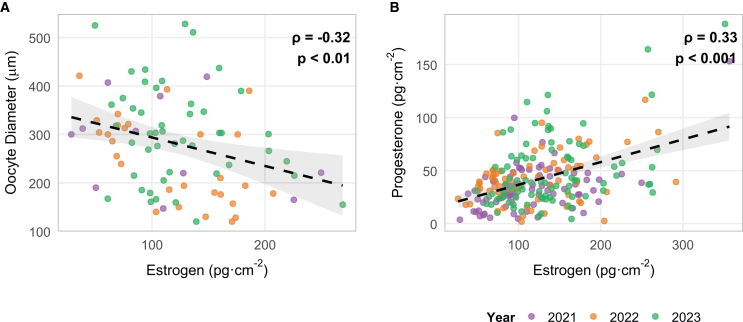
Relationships between steroid hormones and oocyte development in *Acropora eurystoma* (A) Estrogen correlated negatively with oocyte diameter (Spearman’s ρ = −0.32, *p* < 0.01, *n* = 86), indicating hormone decline during maturation. (B) Estrogen and progesterone correlated positively (ρ = 0.33, *p* < 0.001, *n* = 240), suggesting coordinated regulation. Colors denote sampling years (purple: 2021, orange: 2022, green: 2023). Dashed lines: linear regression; gray shading: 95% confidence intervals.

Estrogen showed a significant inverse correlation with oocyte diameter (ρ = −0.32, *p* < 0.01; Figure 2A and Table S7), with hormone levels declining as oocytes matured. Progesterone levels correlated positively with estrogen across all years (ρ = 0.33, *p* < 0.001; Figure 2B and Table S7). Neither steroid hormone correlated significantly with fecundity (Table S7).

#### Environmental correlations with estrogen levels and oocyte development

Photoperiod and UV radiation were stronger predictors of estrogen levels than water temperature (Figure S1). Environmental factors collectively explained 86% of oocyte diameter variance (Table 3; detailed model comparisons in Table S8).

**Table 3:** Environmental factors influencing estrogen levels and oocyte diameter in *Acropora eurystoma*

Environmental Factor	Estrogen Levels				Oocyte Diameter
	2021	2022	2023	All Years Combined	All Years Combined
Photoperiod	37.0%∗	37.5%∗	21.1%∗	32.5%∗	80.1%∗
UV Radiation	22.8%∗	27.1%∗	23.7%∗	26.7%∗	70.1%∗
Water Temperature	18.5%∗	31.5%∗	4.6%	17.9%∗	72.2%∗
PAR	25.1%∗	28.3%∗	22.7%∗	13.2%∗	73.7%∗
Multi-factor Model	–	–	–	36.3%∗	86.2%∗

Values represent percentage of deviance explained by generalized additive models (GAMs). Years were analyzed separately due to different sampling frequency (2021–2022: bi-weekly, *n* = 12 colonies; 2023: monthly, *n* = 8 colonies). A combined analysis is presented for comparison, with oocyte diameter emphasized due to its high temporal consistency across years (85.1–90.4% deviance explained). ∗ = Statistically significant (*p* < 0.05). Complete GAM statistics are provided in Table S8.

### Spatial distribution analysis

**Figure 3:**
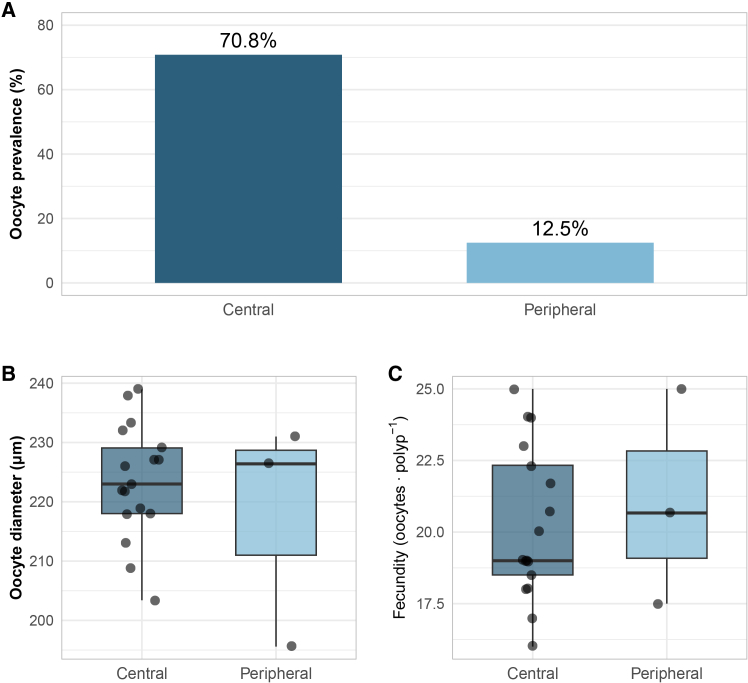
Spatial differences in reproductive parameters within *Acropora eurystoma* colonies (March 2024, *n* = 8) (A) Oocyte prevalence (higher proportion of polyps with oocytes) was higher in central regions (70.8%) than peripheral regions (12.5%, χ^2^ = 14.49, *p* < 0.001). (B) Oocyte diameter and (C) fecundity did not differ between regions (*p* = 0.66, 0.75). Dark blue: central; light blue: peripheral. Boxplots show median, IQR and individual values.

Colonies had surface areas of 4985 ± 490 cm^2^ and volumes of 3253 ± 446 cm^3^ (*n* = 8; Table S4). Central regions had higher proportion of polyps with oocytes (70.8%) compared to peripheral regions (12.5%; Figure 3A) while oocytes diameter (Figure 3B, *p* = 0.66) and the number of oocytes per polyp (fecundity; Figure 3C, *p* = 0.75) did not differ significantly between regions (detailed statistics in Table S9).

**Figure 4:**
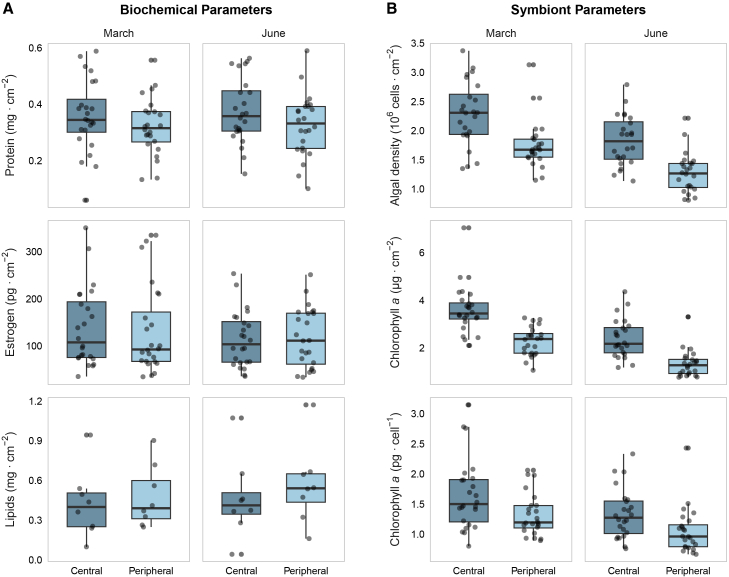
Spatial and temporal variation in physiological traits of *Acropora eurystoma* (A) Protein, estrogen and lipid content showed no central-peripheral differences in March or June 2024 (all *p* > 0.05). (B) Symbiont density and chlorophyll *a* content were higher centrally (all *p* < 0.001) and declined from March to June (algal density: −20%–27%; chlorophyll *a*: −33%–41%). Boxplots: median, IQR, and individual values. *n* = 24 per region (except lipids, *n* = 8).

Estrogen, protein, and lipid concentrations did not differ significantly between central and peripheral regions in either early or late gametogenic stages (Figure 4A and Table 4; detailed statistics in Table S9).

**Table 4:** Summary of spatial and temporal differences in physiological parameters between central and peripheral colony regions of *Acropora eurystoma* colonies

Parameter	Central vs. Peripheral (March)	Central vs. Peripheral (June)	Temporal Change (March to June)
Estrogen levels	NS	NS	NS
Protein concentration	NS	NS	NS
Lipid concentration	NS	NS	NS
Algal cell density	+31.0%∗	+43.8%∗	Central: −20.1%∗ Peripheral: −27.2%∗
Chlorophyll *a* per cm^2^	+58.8%∗	+80.9%∗	Central: −33.3%∗ Peripheral: −41.4%∗
Chlorophyll *a* per algal cell	+25.7%∗	+26.5%∗	Central: −19.4%∗ Peripheral: −19.9%∗
Oocyte prevalence	+466.4%∗	NA	NA
Oocyte size	NS	NA	NA

∗ = *p* < 0.05 indicates statistical significance; NS, Not significant; NA, Not available. Sample sizes were *n* = 24 per group for all parameters except lipids (*n* = 8) and oocytes (central *n* = 17, peripheral *n* = 3 for March data). Detailed spatial statistics are provided in Table S9.

Algal cell density was significantly higher in central areas than peripheral ones (March: 2.29 ± 0.12 vs. 1.75 ± 0.08 million cells/cm^2^, +31.0%. June: 1.83 ± 0.10 vs. 1.27 ± 0.09 million cells/cm^2^, +43.8%). Chlorophyll *a* concentration followed the same trend (March: 3.60 ± 0.21 vs. 2.27 ± 0.12 μg cm^−2^, +58.8%. June: 2.40 ± 0.17 vs. 1.33 ± 0.11 μg cm^−2^, +80.9%; both *p* < 0.001). Both regions showed significant seasonal declines from March to June in algal cell density (central: −20.1%; peripheral: −27.2%) and chlorophyll *a* (central: −33.3%; peripheral: −41.4%, Figure 4B, Tables 4 and S9).

## Discussion

This study examined the temporal and spatial heterogeneity of steroid hormones in *A. eurystoma* in relation to gametogenesis over three consecutive reproductive cycles (2021–2023) to decipher steroids’ role in coral reproductive biology.

### Estrogen dynamics challenge the pre-spawning elevation paradigm

**Figure 5:**
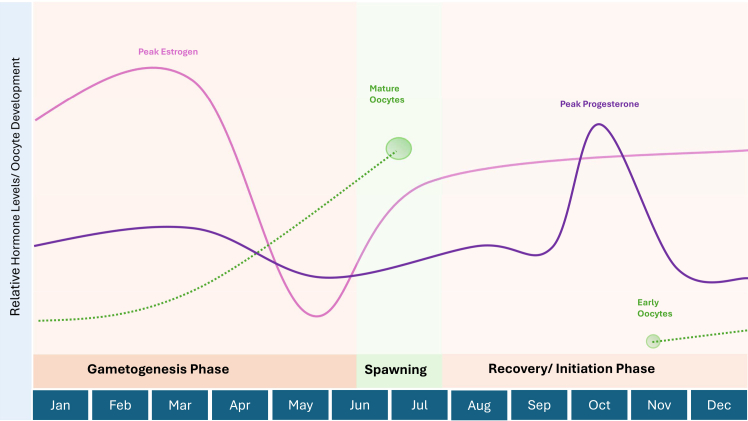
Conceptual model summarizing annual reproductive and hormonal dynamics in *Acropora eurystoma* Estrogen (pink) peaks during early-mid gametogenesis (January-March) and declines with oocytes maturation. Progesterone (purple) remains low during gametogenesis but peaks in October, signaling cycle renewal. Oocyte development (green dashed) follows sigmoidal growth through spawning (Jun-Jul). The cycle comprises three phases: gametogenesis (Jan-Jun), spawning (Jun-Jul), and recovery/initiation (Jul-Dec).

Estrogen consistently peaked in March, declining before spawning, challenging the prevailing model of pre-spawning elevation. While earlier studies^19^^,^^20^ reported elevated estrogen near spawning, our three-year dataset shows that estrogen peaks during early gametogenesis (March), then decreases as oocytes grow and mature (April-May), and recovers around spawning periods (Figure 5). This pattern suggests two non-mutually exclusive mechanisms. First, elevated estrogen during early oogenesis may initiate vitellogenic processes, the nutrient accumulation phase in developing oocytes. The subsequent decline could then signal completion of this preparatory phase and trigger transition to final maturation, similar to vertebrate systems where hormone withdrawal initiates developmental transitions (e.g., the luteinizing hormone surge following estrogen decline). Alternatively, the estrogen decline may reflect metabolic reallocation during the most energetically demanding growth phase. As oocytes enter rapid growth, reducing hormone production could redirect metabolic resources toward oocyte biosynthesis. Both interpretations are supported by the inverse correlation between estrogen and oocyte diameter. Estrogen levels are highest when oocytes are still small and begin nutrient accumulation, then decline as oocytes enlarge and approach maturity. This pattern suggests estrogen acts as an early developmental regulator, rather than a continuous promoter of oocyte growth.

Tan et al.^21^ detected E2 in *Acropora tenuis* during vitellogenic months (March-May) but not post-spawning (June). Similarly, our multi-year study found a comparable pattern over three years, extending this observation to *A. eurystoma* and including progesterone dynamics and quantitative oocyte growth measurements. While these temporal associations observed strongly suggest an endocrine regulatory role, we note that molecular evidence for receptor-mediated signaling remains to be established.

### Progesterone as a cycle initiation signal

Progesterone remained stable during reproductive months but showed a pronounced October peak, three months post-spawning (measured over one complete annual cycle in 2022). This peak may signal the initiation of the next reproductive cycle, echoing patterns in soft corals.^14^ Since gametogenesis onset often begins shortly after spawning in broadcast spawners^4^^,^^10^ the October peak suggests distinct steroid hormones may influence different phases of the coral reproductive cycle. If this October pattern proves consistent across years, a temporal separation between estrogen and progesterone peaks (Figure 5) could suggest a sequential pattern: progesterone may initiate gametogenesis in autumn, followed by estrogen-mediated vitellogenesis in late winter/spring. However, despite their different temporal peaks, progesterone and estrogen showed positive correlation across all sampling points, suggesting coordinated variation even during months when neither shows distinct peaks. This coordination despite temporally distinct functional peaks may reflect shared upstream regulation or interdependent synthesis pathways.

### Oocyte development and environmental regulation

Oocyte diameter showed strong temporal patterns, with environmental factors collectively explaining 86% of variance, substantially more than hormones explained (Table 3). This suggests environmental cues may drive developmental progression,^9^ while steroid hormones coordinate timing of developmental transitions. In contrast, fecundity was independent of steroid hormone levels, suggesting hormones regulate developmental timing rather than oocyte proliferation.

### Light-estrogen correlations during gametogenesis: Implications for climate change

Photoperiod and UV radiation showed stronger statistical associations with estrogen levels than water temperature during the gametogenic period (Table 3). While our measurements captured total UV exposure and broad-spectrum PAR rather than specific wavelengths, the consistent multi-year correlation suggests light-related cues may play important roles in endocrine regulation during gametogenesis. These light-estrogen associations during gametogenesis parallel broader patterns in coral reproduction, where photoperiod and solar insolation can be stronger predictors of spawning timing than temperature,^32^^,^^33^ even though temperature also influences reproductive timing in *Acropora* species.^34^^,^^35^^,^^36^ The mechanisms linking environmental cues to reproductive physiology remain unclear, but light-responsive photoreceptor pathways, including opsins^37^ that mediate spawning in response to lunar cycles^7^ provide a plausible mechanistic link between photoperiod and long-term steroid hormone regulation.

Most research on environmental regulation of coral reproduction has focused on spawning timing, the hours or days surrounding gamete release, rather than the months-long gametogenic period. Potential decoupling between gametogenesis and spawning cues may explain observations of spawning desynchronization and split spawning events documented in some populations.^2^ Experimental evidence^38^ demonstrates that temperature affects both spawning timing and gamete quality: a 2°C increase advanced spawning by one day in *Acropora digitifera* while reducing egg size and sperm number. Similarly, Bouwmeester et al.^39^ showed that both temperature and UV radiation influence spawning timing. However, these studies examined effects during spawning rather than throughout gametogenesis, leaving open the question of whether different environmental cues regulate different phases of reproduction.

Nevertheless, causality cannot be established without experimental manipulation. Experimental manipulation of light and temperature, as demonstrated for spawning,^38^^,^^39^ integrated with hormone monitoring throughout gametogenesis, would clarify whether environmental cues have stage-specific effects on gametogenesis versus spawning. Such work could reveal whether climate-driven changes in light and temperature regimes^2^ pose risks of decoupling gametogenesis from spawning, potentially threatening coral reproductive success.

### Spatial reproductive heterogeneity

While estrogen levels were consistent across colonies, oocyte prevalence was substantially higher in central areas than at the periphery. This suggests that uniform hormone levels alone do not directly determine local reproductive output. Instead, local factors, such as polyp age and energy reserves, modulate how individual polyps respond to endocrine signals.

Spatial heterogeneity in reproductive capacity is well-documented in coral colonies, with central regions showing enhanced reproductive output in branching *Acropora* species.^23^ Central polyps are typically older and likely possess greater energy reserves than younger peripheral polyps.^24^^,^^25^ This hierarchical regulation model has important implications for understanding coral reproductive biology and suggests new research directions investigating hormone receptor expression, local signaling pathways, and the physiological factors that determine reproductive competence at the polyp level.

### Conservation and restoration implications

Our findings offer a physiological framework for detecting early signs of reproductive disruption in corals. Hormonal shifts may serve as sensitive indicators of environmental stress, preceding observable changes in gamete development or spawning. The progesterone peak at gametogenesis onset (October) and the estrogen peak during early vitellogenesis (March) could serve as biomarkers for reproductive health: deviations from these patterns may indicate disrupted gametogenesis before spawning occurs.

From a restoration perspective, the 5.7-fold difference in oocyte prevalence (higher proportion of polyps with oocytes) between central and peripheral regions suggests that fragment location affects reproductive capacity. Prioritizing central regions as donor material may enhance reproductive output in restored colonies.^23^^,^^24^

Our multi-year study demonstrates clear seasonal dynamics in estrogen and progesterone in *A. eurystoma*. Estrogen peaks in March followed by declines during oocyte maturation challenge the prevailing paradigm of pre-spawning elevation, while a progesterone peak in October suggests a role in gametogenesis initiation. Photoperiod and UV radiation emerged as stronger predictors of estrogen levels than temperature during gametogenesis, suggesting that light-related cues may influence coral endocrine dynamics. Despite uniform hormone distribution, spatial heterogeneity in oocyte prevalence reveals that local polyp-level factors modulate colony-wide endocrine signals. These findings position hormonal profiling as a potential early-warning system for detecting reproductive disruption.

### Limitations of the study

This study focused on *A. eurystoma* at one location in the Gulf of Aqaba/Eilat, enabling detailed multi-year temporal tracking. Extending this approach to additional coral species and geographic regions would help determine whether these hormonal patterns are widespread across corals. Future experimental studies could directly test causal mechanisms underlying the temporal correlations we observe. Hormone manipulations (aromatase inhibitors, exogenous supplementation) could determine if estrogen directly regulates oocyte growth. Controlled light experiments could test whether photoperiod and UV regulate hormone synthesis. Temperature manipulations could distinguish effects on gametogenesis versus spawning timing. Molecular approaches including receptor identification, steroidogenic enzyme profiling, and gene expression analyses could establish mechanistic pathways linking environmental cues to reproductive output.

## Resource availability

### Lead contact

Requests for further information and resources should be directed to and will be fulfilled by the lead contact, Chen Azulay (chen.azulay1@mail.huji.ac.il).

### Materials availability

This study did not generate new materials or reagents.

### Data and code availability

#### Data

Data reported in this paper will be shared by the lead contact upon request.

#### Code

Code used for data analysis is openly available on Github: https://github.com/AzulayChen/Steroid-hormones-dynamics-in-Acropora-eurystoma-.git.

#### Other items

Any additional information required to reanalyze the data reported in this paper is available from the lead contact upon request.

## Acknowledgments

We are grateful to Naama Rose Kochman, Dror Komet, Keren-Or Rinkov, Dr. Marleen Stuhr, Alessandra Pak, and Britt Ronen for their invaluable assistance with field sampling. We thank Dr. Anne McElroy for contributing to experimental design, Dr. Carrie McDonough for LC-MS analysis of seawater samples at 10.13039/100007259Stony Brook University, and Dr. Julia Cerutti for photogrammetry and 3D modeling. We thank the National Monitoring Program of the Gulf of Eilat for environmental data. We thank the 10.13039/100020704Inter-University Institute for Marine Sciences in Eilat for logistical support. This study was funded by the 10.13039/100000001National Science Foundation (NSF-BSF 878 research grant program) under grant no. 2023684.

## Author contributions

writing – original draft, visualization, validation, methodology, investigation, formal analysis, data curation, conceptualization, C.A.; Writing – review and editing, supervision, resources, funding acquisition, conceptualization, K.K.; Writing – review and editing, supervision, resources, funding acquisition, conceptualization, M.F.

## Declaration of interests

The authors declare no competing interests.

## STAR★Methods

### Key resources table

**Table:** 

REAGENT or RESOURCE	SOURCE	IDENTIFIER
**Biological samples**		

*Acropora eurystoma*	Gulf of Aqaba, Eilat, Northern Red Sea (29.30°*N* 34.55°E)	Nature and Park Authority, Israel, permit no.:2021/42769, 2023/43329,2024/43531

**Chemicals, peptides, and recombinant proteins**		

Estrogen ELISA Kit	Eagle Biosciences	ESG31-KO1
Progesterone ELISA Kit	ALPCO	11-PROHU-E01-SLV
Diethyl ether	Sigma-Aldrich	309966
Phosphate-buffered saline	Sigma-Aldrich	P3813
Acetone	Sigma-Aldrich	STBJ2516
Formaldehyde solution	Sigma	MKCQ6645
Formic acid	Sigma-Aldrich	F0507
Sodium citrate	Fisher Chemical	S/3320/60
Bradford protein assay	Bio-Rad	5000205

**Software and algorithms**		

R	R Core Team	version 4.5.0
ggplot2 package	Wickham, 2016	Version 4.0/1
mgcv package	Wood, 2011	Version 1.9–4
suncalc package	Thieurmel & Elmarhraoui	Version 0.5.1
ImageJ	ImageJ GitHub	https://imagej.nih.gov/ij/
Agisoft Metashape Professional	Agisoft LLC	Version 2.0.2

**Deposited data**		

Raw data and R scripts	This paper	https://github.com/AzulayChen/Steroid-hormones-dynamics-in-Acropora-eurystoma-.git

**Other**		

ChemCatcher C-18 disks	Supelco	SU66883-U
Plate reader	BioTek	HT Synergy
Stereoscope	Leica Microsystems	M165 FC

### Experimental model and study participant details

The study organism was *Acropora eurystoma*, a common broadcast-spawning scleractinian coral in the Gulf of Aqaba (GoA), northern Red Sea. This species is hermaphroditic and exhibits annual reproductive cycles with spawning typically occurring in June-July in the GoA. Colonies were maintained in a coral nursery under natural environmental conditions. All procedures were approved by the Nature and Park Authority, Israel (permits no.: 2021/42769, 2023/43329, 2024/43531).

### Method details

#### Study site and sample collection

Coral fragments were collected from pre tagged 12 *A. eurystoma* colonies (>20 cm diameter) at a depth of 7–8 m in a coral nursery located in the northern GoA, near the Interuniversity Institute for Marine Sciences in Eilat, Israel (IUI; 29°30′N, 34°55′E). The site is characterized by oligotrophic waters, fringing coral reefs, and is within a marine nature reserve with relatively stable environmental conditions and limited anthropogenic disturbance.

Sampling was conducted during the months preceding spawning in the GoA^2^^,^^10^ across three consecutive reproductive cycles. In 2021 and 2022, bi-weekly sampling from January through July was conducted, alternating between two groups of six colonies, so that each of the 12 colonies was sampled once per month to reduce excessive stress on any single colony during gametogenesis. In 2023, monthly sampling from January through July was conducted on eight colonies as four colonies showed signs of stress during 2022 and sampling was discontinued. The eight remaining colonies showed no signs of stress despite maintaining similar sampling frequency, indicating minimal impact from repeated sampling.

To establish a baseline hormone level outside of the gametogenic cycle, when oocytes are typically absent,^26^ additional sampling was performed from August to November in 2022 only (herein “non-reproductive” period) following the same protocol for hormone quantification and oocyte examination. For collection dates see Table S1.

At each sampling point, two fragments (3–4 cm in length) were collected from each colony. One fragment was fixed in 10% formalin in seawater for 24 h, then washed with fresh water and preserved in 70% ethanol for gametogenesis progression analysis, while the second fragment was snap-frozen in liquid nitrogen and stored at −80°C for subsequent steroid hormone extraction and quantification. Detailed sampling frequency per colony is provided in Table S2.

#### Temporal patterns in hormone levels

##### Chemical analysis of seawater *in situ*

To establish baseline environmental hormone levels and confirm the study site is suitable for detecting endogenous coral hormone production, we measured dissolved steroid hormones in seawater using passive samplers. In 2021, ChemCatcher (with C-18 disks) were deployed next to the coral nursery in triplicates and replaced monthly over a six-month period (January-June). Three additional groups of triplicate samplers were deployed in the same site for durations of two, three, and eight weeks to assess optimal deployment time for hormone detection (Table S3) The limit of quantitation (LOQ) was 0.125 ng E2 per disk, corresponding to 0.015–0.058 ng/L for 28-day deployments based on literature sampling rates (0.077–0.302 L/day^40^). Background estrogen concentrations over coral reefs and in uncontaminated marine waters typically range from below detection to ∼1 ng/L.^41^^,^^42^ The samplers were placed in a stainless-steel frame to maintain orientation and protect them from sunlight exposure. After retrieval, samplers were wrapped in aluminum foil and stored at −20°C until lyophilization. The lyophilized samples were shipped on ice to Stony Brook University, where it was spiked with 13C-estradiol (E2) as internal standards and extracted first in methanol and then with methanol: acetone (1:1 v/v) using ultrasonication. Extraction solvents were combined and concentrated under nitrogen for analysis using a 1290 Agilent Infinity ultra-high-pressure liquid chromatography (UPLC) system coupled to an Agilent 6545 quadrupole-time-of-flight-mass spectrometer (QTOF-MS) with negative electrospray ionization.

#### Steroid hormone quantification in coral tissue

Tissue from coral fragments was removed using an airbrush with 2.5 mL of ice-cold phosphate-buffered saline (PBS, pH 7.0) and removed into Ziplock bags. The tissue was homogenized using a manual glass homogenizer, followed by centrifugation at 2,000 g for 5 min at 4°C to separate coral host tissue from symbiotic algae.

Steroids were extracted from the host tissue sample by adding an equal volume of diethyl ether (1:1) and vortex mixing for 60 s. The mixture was then subjected to freezing for 30 min at −80°C to facilitate the separation of the organic phase containing the steroid hormones. This extraction procedure was repeated twice. The organic phase was transferred to clean tubes, aliquoted to cryotubes and stored at −80°C until further analysis. The ether fractions were evaporated to dryness, and the residue was reconstituted in 500 μL of PBS.

Steroid hormone levels were quantified using commercial enzyme-linked immunosorbent assay (ELISA) kits. Total estrogen (100% specificity for E1, E2 and E3) was measured using an Eagle Biosciences kit (cat. ESG31-KO1), and progesterone was measured with an ALPCO direct saliva kit (cat. 11-PROHU-E01-SLV). ELISA plates were prepared according to the manufacturer’s instructions, and absorbance was read at 450 nm using a Biotek HT Synergy plate reader. Hormone concentrations were determined based on standard curves, ensuring that control samples (blanks, low, and high controls) were within acceptable ranges. Extraction efficiency was validated by spiking coral tissue samples with known concentrations of kit standards, followed by the complete extraction protocol. Recovery rates confirmed the reliability of the extraction method for coral tissue matrices. Hormone concentrations were normalized to coral fragment surface area, determined using the single wax-dipping method,^43^ and expressed as pg/cm^2^.

#### Gametogenesis progress and fecundity

The fixed coral fragments designated for gametogenesis analysis were decalcified overnight in 50% formic acid and 20% sodium citrate (1:1 v/v).^44^ The coral tissue was rinsed with distilled water and dissected under a stereoscope (Leica M165 FC). Five polyps at least 2 cm from the fragment tip were randomly selected for further dissection. The number of oocytes per polyp was also recorded to assess fecundity. Maximum diameter of oocytes was measured from images of dissected polyps taken with a millimetric scale and analyzed using ImageJ software.^45^ Oocyte developmental stages were classified following established criteria for *Acropora tenuis*^46^: Stage I (35–75 μm), Stage II (76–175 μm), Stage III (176–285 μm), Stage IV (286–385 μm), and Stage V (>386 μm), providing a comparative framework for *A. eurystoma*.

#### Environmental data collection

Environmental parameters including water temperature, photosynthetically active radiation (PAR), and ultraviolet (UV) radiation were obtained from the National Monitoring Program of the Gulf of Eilat (NMP, https://www.meteo-tech.co.il/eilat-yam/eilat_en.asp) database. The NMP station is located ca. 50 m away from the coral nursery at 2 m depth. Photoperiod (daylight hours) was calculated as the time interval between sunrise and sunset for each sampling date, expressed in decimal hours using the “suncalc” package (v 0.5.1) in R (R Core Team, 2025) which computes sunrise and sunset times based on site coordinates (29°30′N, 34°55′E) and date.

#### Spatial distribution analysis: Central vs. peripheral areas

During 2021–2023, all sampling was conducted from central colony regions to assess temporal hormone dynamics. To investigate spatial variation in steroid hormone levels within colonies, in 2024 each of the eight *A. eurystoma* was divided into two regions: peripheral areas (outermost branches) and central areas (inner regions closest to the vertical growth axis). Colonies were photographed underwater with an Olympus TG-5 camera and artificial lighting (225–340 overlapping images per colony), and surface area was extracted directly from the 3D mesh models. Colony surface area and volume (Table S4) were measured using structure-from-motion photogrammetry (Agisoft Metashape Professional v. 2.0.2). Sampling was conducted at two key timepoints: March (mid-gametogenesis, when oocytes were present) and June (late gametogenesis/pre-spawning).

At each timepoint, three fragments per region were sampled to assess steroid hormone levels and physiological parameters following protocols routinely performed in our lab.^47^ Briefly, tissue was removed and homogenized as described above for hormone extraction. From the homogenized tissue, subsamples were taken before centrifugation for total protein (Bradford assay,^48^ bovine serum albumin standard), total lipid (sulfur-phospho-vanillin method,^49^ corn oil standard), symbiont density (CellDrop Denovix cell counter after 10% formalin preservation), and chlorophyll *a* (acetone extraction, spectrophotometric quantification^50^). All parameters were normalized to coral surface area (single wax-dipping method^43^). Another three fragments were sampled for gametogenesis analysis, as described above.

### Quantification and statistical analysis

Data was analyzed using R (4.5.0, R Core Team, 2025). All variables were tested for normality (Shapiro-Wilk test) and homogeneity of variances (Levene’s test), with non-parametric methods employed when assumptions were violated. Statistical significance was set at *p* < 0.05. Figures were created using “ggplot2”.

In order to assess temporal patterns and environmental relationships, generalized additive models (GAMs) were fitted separately for each year using the “mgcv” package due to different sampling frequencies between years (bi-weekly in 2021–2022, monthly in 2023). Colony identity was included as a random effect using s(colony, bs = “re”) to account for repeated sampling (detailed model outputs in Table S5). Model assumptions were verified using gam.check() diagnostics including Q-Q plots for normality assessment and residual plots for homoscedasticity. Environmental factors (photoperiod, UV radiation, temperature, PAR) were analyzed using combined datasets with year as a covariate.

Temporal comparisons of estrogen levels between sampling points within each year were performed using Kruskal-Wallis tests, followed by post-hoc pairwise comparisons using Dunn’s tests with Bonferroni correction for multiple comparisons.

Spatial differences in physiological parameters between central and peripheral colony regions were analyzed using Welch’s t-tests. two-way ANOVA was employed to test for location effects, temporal effects (March vs. June), and location-by-time interactions. Post-hoc comparisons were conducted using Tukey’s HSD test. For oocyte prevalence (proportion of polyps containing visible oocytes), comparisons between central and peripheral regions were performed using chi-square tests. Spatial and temporal patterns in algal cell density, chlorophyll content, and other physiological parameters were analyzed using linear mixed models, with location and month as fixed factors.

Relationships between estrogen and progesterone levels and reproductive parameters (oocyte diameter and fecundity) were assessed using Spearman’s rank correlation on pooled data from all years (2021–2023).

## References

[1] Hughes T.P., Anderson K.D., Connolly S.R., Heron S.F., Kerry J.T., Lough J.M., Baird A.H., Baum J.K., Berumen M.L., Bridge T.C. (2018). Spatial and temporal patterns of mass bleaching of corals in the Anthropocene. Science.

[2] Shlesinger T., Loya Y. (2019). Breakdown in spawning synchrony: A silent threat to coral persistence. Science.

[3] Olischläger M., Wild C. (2020). How does the sexual reproduction of marine life respond to ocean acidification?. Diversity.

[4] Harrison P.L., Wallace C. (1990). Reproduction, dispersal and recruitment of scleractinian corals Ecosystems of the world. 25: Coral Reefs. Ecosyst World 25 Coral Reefs.

[5] Baird A.H., Guest J.R., Willis B.L. (2009). Systematic and biogeographical patterns in the reproductive biology of scleractinian corals. Annu. Rev. Ecol. Evol. Syst..

[6] Babcock R.C., Bull G.D., Harrison P.L., Heyward A.J., Oliver J.K., Wallace C.C., Willis B.L. (1986). Synchronous spawnings of 105 scleractinian coral species on the Great Barrier Reef. Mar. Biol..

[7] Kaniewska P., Alon S., Karako-Lampert S., Hoegh-Guldberg O., Levy O. (2015). Signaling cascades and the importance of moonlight in coral broadcast mass spawning. eLife.

[8] Keith S.A., Maynard J.A., Edwards A.J., Guest J.R., Bauman A.G., van Hooidonk R., Heron S.F., Berumen M.L., Bouwmeester J., Piromvaragorn S. (2016). Coral mass spawning predicted by rapid seasonal rise in ocean temperature. Proc. R. Soc. A B..

[9] Wuitchik D.M., Wang D., Pells T.J., Karimi K., Ward S., Vize P.D. (2019). Seasonal temperature, the lunar cycle and diurnal rhythms interact in a combinatorial manner to modulate genomic responses to the environment in a reef-building coral. Mol. Ecol..

[10] Shlesinger Y., Goulet T.L., Loya Y. (1998). Reproductive patterns of scleractinian corals in the northern Red Sea. Mar. Biol..

[11] Meccariello R., Fasano S., Pierantoni R., Cobellis G. (2014). Modulators of Hypothalamic–Pituitary–Gonadal Axis for the Control of Spermatogenesis and Sperm Quality in Vertebrates. Front. Endocrinol..

[12] Whirledge S., Cidlowski J.A. (2019). Steroid Hormone Action. Yen Jaffe’s Reprod Endocrinol Physiol Pathophysiol Clin Manag Eighth Ed..

[13] Barbaglio A., Sugni M., Di Benedetto C., Bonasoro F., Schnell S., Lavado R., Porte C., Candia Carnevali D.M. (2007). Gametogenesis correlated with steroid levels during the gonadal cycle of the sea urchin Paracentrotus lividus (Echinodermata: Echinoidea). Comp. Biochem. Physiol. Part A Mol Integr Physiol..

[14] Slattery M., Hines G., Starmer J., Paul V. (1999). Chemical signals in gametogenesis, spawning, and larval settlement and defense of the soft coral Sinularia polydactyla. Coral Reefs.

[15] Lafont R., Mathieu M. (2007). Steroids in aquatic invertebrates. Ecotoxicology.

[16] Tarrant A.M., Atkinson S., Atkinson M.J. (1999). Estrone and estradiol-17 beta concentration in tissue of the scleractinian coral, Montipora verrucosa. Comp. Biochem. Physiol. Mol. Integr. Physiol..

[17] Twan W.H., Hwang J.S., Lee Y.H., Wu H.F., Tung Y.H., Chang C.F. (2006). Hormones and reproduction in scleractinian corals. Comp. Biochem. Physiol. Mol. Integr. Physiol..

[18] Rougée L.R.A., Richmond R.H., Collier A.C. (2015). Molecular reproductive characteristics of the reef coral Pocillopora damicornis. Comp. Biochem. Physiol. -Part A Mol. Integr. Physiol..

[19] Atkinson S., Atkinson M.J. (1992). Detection of estradiol-17β during a mass coral spawn. Coral Reefs.

[20] Twan W.H., Hwang J.S., Chang C.F. (2003). Sex steroids in scleractinian coral, Euphyllia ancora: Implication in mass spawning. Biol. Reprod..

[21] Tan E.S., Hamazato H., Ishii T., Taira K., Takeuchi Y., Takekata H., Isomura N., Takemura A. (2021). Does estrogen regulate vitellogenin synthesis in corals?. Comp. Biochem. Physiol. -Part A Mol. Integr. Physiol..

[22] Tarrant A.M., Atkinson M.J., Atkinson S. (2004). Effects of steroidal estrogens on coral growth and reproduction. Mar. Ecol. Prog. Ser..

[23] Soong K., Lang J.C. (1992). Reproductive integration in reef corals. Biol. Bull..

[24] Nozawa Y., Lin C.H. (2014). Effects of colony size and polyp position on polyp fecundity in the scleractinian coral genus Acropora. Coral Reefs.

[25] Hall V.R., Hughes T.P. (1996). Reproductive strategies of modular organisms: Comparative studies of reef-building corals. Ecology.

[26] Shlesinger Y., Loya Y. (1985). Coral community reproductive patterns: Red Sea versus the Great Barrier Reef. Science (80- ).

[27] Fine M., Gildor H., Genin A. (2013). A coral reef refuge in the Red Sea. Glob. Chang. Biol..

[28] Bellworthy J., Fine M. (2017). Beyond peak summer temperatures, branching corals in the Gulf of Aqaba are resilient to thermal stress but sensitive to high light. Coral Reefs.

[29] Krueger T., Horwitz N., Bodin J., Giovani M.E., Escrig S., Meibom A., Fine M. (2017). Common reef-building coral in the northern red sea resistant to elevated temperature and acidification. R. Soc. Open Sci..

[30] Kochman N.R., Fine M. (2025). Science of the Total Environment Gulf of Aqaba as a thermal refuge : Insights from four years of intensifying marine heatwaves. Sci. Total Environ..

[31] Osman E.O., Smith D.J., Ziegler M., Kürten B., Conrad C., El-Haddad K.M., Voolstra C.R., Suggett D.J. (2018). Thermal refugia against coral bleaching throughout the northern Red Sea. Glob. Chang. Biol..

[32] Van Woesik R., Lacharmoise F., Köksal S. (2006). Annual cycles of solar insolation predict spawning times of Caribbean corals. Ecol. Lett..

[33] Penland L., Kloulechad J., Idip D., Van Woesik R. (2004). Coral spawning in the western Pacific Ocean is related to solar insolation: Evidence of multiple spawning events in Palau. Coral Reefs.

[34] Osman E.O., Suggett D.J., Attalla T.M., Casartelli M., Cook N., El-Sadek I., Gallab A., Goergen E.A., Garcias-Bonet N., Glanz J.S. (2024). Spatial variation in spawning timing for multi-species Acropora assemblages in the Red Sea. Front. Mar. Sci..

[35] Sakai Y., Hatta M., Furukawa S., Kawata M., Ueno N., Maruyama S. (2020). Environmental factors explain spawning day deviation from full moon in the scleractinian coral Acropora. Biol. Lett..

[36] Yoshioka Y., Suzuki G., Fujikura Y., Tashiro S., Uchida T. (2025). Time- - Series RNA- - Seq of Acropora tenuis Reveals Molecular Waves Leading to Synchronous Mass Spawning of Scleractinian Corals. Mol. Ecol..

[37] Shi Z., Takemura A. (2025). Interplay between light and circadian rhythms in the regulation of photoreception and physiological processes in the stony coral Acropora digitifera. Front. Mar. Sci..

[38] Paxton C.W., Baria M.V.B., Weis V.M., Harii S. (2016). Effect of elevated temperature on fecundity and reproductive timing in the coral Acropora digitifera. Zygote.

[39] Bouwmeester J., Daly J., Zuchowicz N., Lager C., Henley E.M., Quinn M., Hagedorn M. (2023). Solar radiation, temperature and the reproductive biology of the coral Lobactis scutaria in a changing climate. Sci. Rep..

[40] Kuster M., Cal A.D., Eljarrat E., Alda M.J.L.D., Barceló D. (2010). Talanta Evaluation of two aquatic passive sampling configurations for their suitability in the analysis of estrogens in water. Talanta.

[41] Tarrant A.M., Atkinson M.J., Atkinson S. (2001). Uptake of estrone from the water column by a coral community. Mar. Biol..

[42] Armoza-Zvuloni R., Kramarsky-Winter E., Rosenfeld H., Shore L.S., Segal R., Sharon D., Loya Y. (2012). Reproductive characteristics and steroid levels in the scleractinian coral Oculina patagonica inhabiting contaminated sites along the Israeli Mediterranean coast. Mar. Pollut. Bull..

[43] Veal C.J., Carmi M., Fine M., Hoegh-Guldberg O. (2010). Increasing the accuracy of surface area estimation using single wax dipping of coral fragments. Coral Reefs.

[44] Rinkevich B., Loya Y. (1979). The Reproduction of the Red Sea Coral Stylophora pistillata. I. Gonads and Planulae. Mar. Ecol. Prog. Ser..

[45] Abràmoff M.D., Magalhães P.J., Ram S.J. (2004). Image processing with ImageJ. Biophotonics Int.

[46] Tan E.S., Izumi R., Takeuchi Y., Isomura N., Takemura A. (2020). Molecular approaches underlying the oogenic cycle of the scleractinian coral, Acropora tenuis. Sci. Rep..

[47] Kochman-Gino N.R., Fine M. (2023). Reef building corals show resilience to the hottest marine heatwave on record in the Gulf of Aqaba. Front. Mar. Sci..

[48] Bradford M.M. (1976). A rapid and sensitive method for the quantitation of microgram quantities of protein utilizing the principle of protein-dye binding. Anal. Biochem..

[49] Cheng Y.-S., Zheng Y., VanderGheynst J.S. (2011). Rapid quantitative analysis of lipids using a colorimetric method in a microplate format. Lipids.

[50] Jeffrey S.W., Humphrey G.F. (1975). New spectrophotometric equations for determining chlorophylls a, b, c1 and c2 in higher plants, algae and natural phytoplankton. Biochem. Physiol. Pflanz..

