# Supplementary material for: Steroid hormones dynamics during coral reproduction: Multi-year patterns in Acropora eurystoma from the Red Sea
Source: iScience. 2026 Jun 2;29(6):116205. doi: 10.1016/j.isci.2026.116205 (PMC13254851; doi:10.1016/j.isci.2026.116205)
Supplement: Document S1. Figure S1, and Tables S1–S9 [file mmc1.pdf]

iScience, Volume *29*

Supplemental information

Steroid hormones dynamics during coral reproduction: Multi-year patterns in *Acropora eurystoma* from the Red Sea

Chen Azulay, Karine Kleinhaus, and Maoz Fine

Supplementary Information

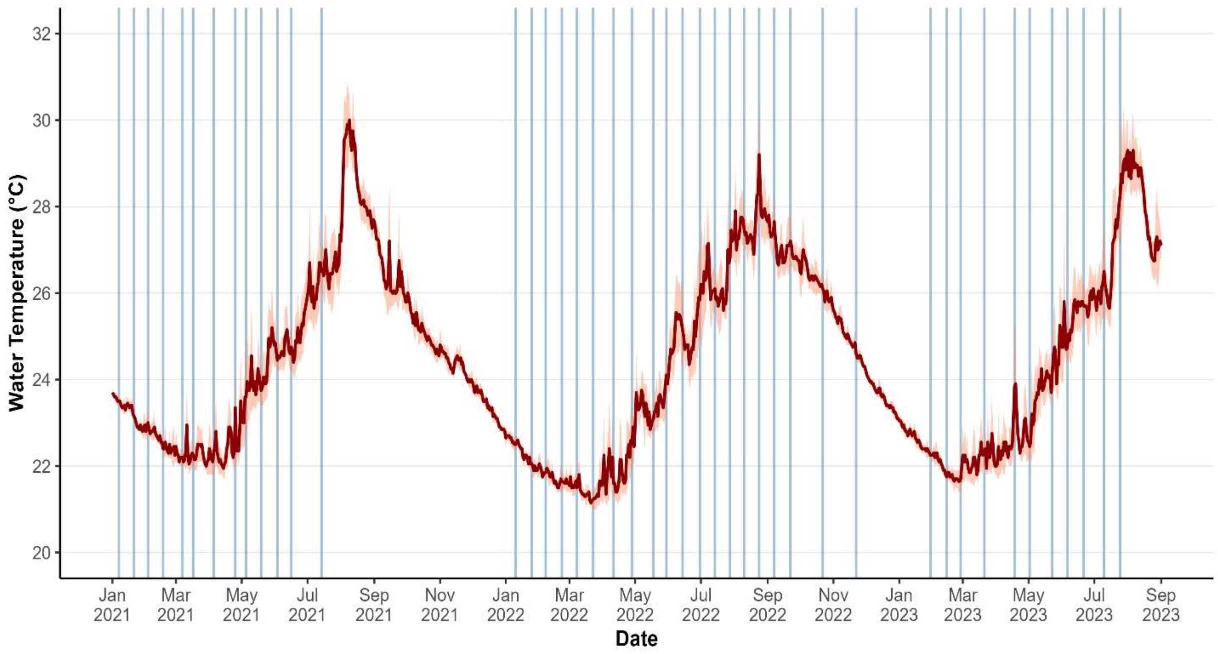

Figure S1. Seawater temperature variability throughout the study period

Water temperature at the study site (Northern Gulf of Aqaba, 29°30′N, 34°55′E) from January 2021 to September 2023, obtained from the National Monitoring Program database. Shaded area indicates daily minimum-maximum range; red line represents daily mean. Vertical blue lines denote coral sampling dates (I=44 sampling events).

Table S1. Coral tissue sampling schedule

Dates of coral fragment collection from tagged *Acropora eurystoma* during three reproductive seasons (2021–2023).

Sampling Point	Date	Sampling Point	Date
**1**	07/01/2021	23	30/05/2022
**2**	21/01/2021	24	13/06/2022
**3**	03/02/2021	25	30/06/2022
**4**	17/02/2021	26	14/07/2022
**5**	07/03/2021	27	28/07/2022
**6**	17/03/2021	28	10/08/2022
**7**	05/04/2021	29	24/08/2022
**8**	25/04/2021	30	07/09/2022
**9**	05/05/2021	31	22/09/2022
**10**	19/05/2021	32	22/10/2022
**11**	03/06/2021	33	22/11/2022
**12**	16/06/2021	34	30/01/2023
**13**	14/07/2021	35	14/02/2023
**14**	10/01/2022	36	27/02/2023
**15**	25/01/2022	37	21/03/2023
**16**	07/02/2022	38	18/04/2023
**17**	22/02/2022	39	02/05/2023
**18**	08/03/2022	40	23/05/2023
**19**	23/03/2022	41	06/06/2023
**20**	11/04/2022	42	21/06/2023
**21**	28/04/2022	43	10/07/2023
**22**	18/05/2022	44	25/07/2023

Table S2. Colony sampling frequency across three reproductive seasons

Number of times each *Acropora eurystoma* colony was sampled during the three-year study period (2021-2023). Each sampling event involved collection of two fragments per colony: one for histological analysis and one for hormone quantification.

Colony	2021	2022	2023	Total
**1**	7	11	11	29
**2**	7	11	11	29
**3**	7	6	NA	13
**4**	6	9	NA	15
**5**	7	5	NA	12

**6**	6	11	11	28
**7**	7	11	11	29
**8**	7	11	11	29
**9**	7	11	10	28
**10**	7	4	NA	11
**11**	7	11	11	29
**12**	7	11	11	29

Table S3. Passive sampler deployments and estradiol (E2) detection in seawater

Deployment schedule and estradiol (E2) detection results from ChemCatcher® passive samplers (C18 disks, PES membrane) deployed in 2021. Reported water concentrations are approximate, derived from published E2 sampling rates (0.077–0.302 L day^{-1}) and deployment duration. No E2 was detected in laboratory blanks. The limit of quantitation (LOQ): 0.125 ng per disk, corresponding to 0.015-0.058 ng/L for 28-day deployments. The concentration range reflects variation in literature-reported sampling rates (0.077-0.302 L/day; Kuster et al., 2010), with lower concentrations calculated assuming higher flow rates and greater water volume sampled. The absence of E2 in most samplers indicates average concentrations < 0.058 ng L^{-1}, validating the site as suitable for endogenous hormone analysis.

Group	Year	Deployment Date	Recovery Date	Duration (days)	E2 on Disk (ng)	Volume Sampled (L)*	Estimated Water Conc. (ng/L)*
**1**	2021	7 Jan	21 Jan	14	2.72	1.08 - 4.23	0.64 - 2.53
**2**	2021	7 Jan	28 Jan	21	2.58	1.62 - 6.34	0.41 - 1.60
**3**	2021	7 Jan	4 Feb	28	1.47	2.16 - 8.46	0.17 - 0.68
**4**	2021	7 Jan	4 Feb	56	0.00	4.31 - 16.91	< LOQ
**5**	2021	4 Feb	4 Mar	28	0.00	2.16 - 8.46	< LOQ
**6**	2021	4 Mar	1 Apr	28	0.81	2.16 - 8.46	0.10 - 0.37
**7**	2021	1 Apr	29 Apr	28	0.00	2.16 - 8.46	< LOQ
**8**	2021	29 Apr	27 May	28	0.00	2.16 - 8.46	< LOQ

**9**	2021	27 May	24 Jun	28	0.00	2.16 - 8.46	< LOQ

Table S4. Colony morphometrics

Surface area and volume of *Acropora eurystoma* colonies used for spatial distribution analysis (March 2024).

**Colony**	**Surface Area (cm^2)**	**Volume (cm^3)**
**1**	5,551.31	3,350
**2**	5,750.52	3,659
**6**	5,040.67	3,183
**7**	2,506.04	1,279
**8**	7,298.79	5,837
**9**	4,316.57	2,894
**11**	4,229.12	2,807
**12**	5,185.42	3,016

Table S5. Generalized Additive Model (GAM) results for temporal patterns

GAMs were fitted separately by year due to different sampling frequencies (2021-2022 = bi-weekly; 2023 = monthly). Combined models include colony as a random effect. k = basis dimension, EDF = effective degrees of freedom, GCV = generalized cross-validation score, AIC = Akaike Information Criterion. *p < 0.05; **p < 0.01; ***p < 0.001; NS = not significant.

Parameter	Year	Period	Formula	N	k_value	EDF	Deviance_Explained	R_squared	GCV	AIC	p_value	Significance
**Estrogen**	2021	Reproductive	Estrogen_sa ~ s(sampling_point, bs = 'cs', k = 8)	82	8	5.59	39.61886	0.351391	433.3729	858.6947	1.41011228038668e-06	***
**Estrogen**	2022	Reproductive	Estrogen_sa ~ s(sampling_point, bs = 'cs', k = 8)	72	8	3.59	43.90505	0.409176	377.6635	757.2631	0	***
**Estrogen**	2023	Reproductive	Estrogen_sa ~ s(sampling_point, bs = 'cs', k = 8)	93	8	4.33	26.32668	0.226875	500.6567	1001.144	5.42172071316127e-05	***
**Estrogen**	Combined	Reproductive	Estrogen_sa ~ s(sampling_point, bs = 'cs', k = 10) + s(colony, bs = 're') + factor(year)	247	10	6.79	24.19436	0.213854	1320.646	2648.348	0	***
**Progesterone**	2021	Reproductive	Progesterone_sa ~ s(sampling_point, bs = 'cs', k = 8)	78	8	0	0.004035	1.74414990344873e-05	349.6484	706.9624	0.352044	NS
**Progesterone**	2022	Reproductive	Progesterone_sa ~ s(sampling_po	70	8	0	9.8556631049908e-05	-4.33E-06	315.424	638.6746	0.834212	NS

			int, bs = 'cs', k = 8)									
**Progesterone**	2023	Reproductive	Progesterone_sa ~ s(sampling_point, bs = 'cs', k = 8)	92	8	0	0.000131	-2.78E-06	449.6823	907.6718	0.775686	NS
**Progesterone**	2022	All Months	Progesterone_sa ~ s(sampling_point, bs = 'cs', k = 8)	93	8	2.25	27.59873	0.257858	449.0093	902.7872	7.13486922876427e-07	***
**Progesterone**	Combined	All/Repro	Progesterone_sa ~ s(sampling_point, bs = 'cs', k = 15) + s(colony, bs = 're') + factor(year)	263	15	4.48	17.68693	0.155987	1259.678	2532.403	2.5382500903337e-06	***
**Oocyte Diameter**	2021	Reproductive	Diameter ~ s(sampling_point, bs = 'cs', k = 5)	11	5	1.91	90.41677	0.881477	53.48017	113.6544	5.28529867562799e-05	***
**Oocyte Diameter**	2022	Reproductive	Diameter ~ s(sampling_point, bs = 'cs', k = 8)	28	8	2.25	84.68373	0.832889	140.2953	286.5826	0	***
**Oocyte Diameter**	2023	Reproductive	Diameter ~ s(sampling_point, bs = 'cs', k = 8)	47	8	2.97	88.11947	0.87299	236.4459	476.5433	0	***
**Oocyte Diameter**	Combined	Reproductive	Diameter ~ s(sampling_point, bs = 'cs', k = 10) + s(colony, bs =	86	10	4.81	87.63645	0.864699	428.4241	874.2473	0	***

			're') + factor(year)									
**Fecundity**	2021	Reproductive	Fecundity ~ s(sampling_point, bs = 'cs', k = 5)	11	5	0.66	20.18493	0.145668	25.81027	57.08867	0.137858	NS
**Fecundity**	2022	Reproductive	Fecundity ~ s(sampling_point, bs = 'cs', k = 8)	28	8	0	3.37305926782218e-05	-1.15E-06	53.26764	110.0072	0.791827	NS
**Fecundity**	2023	Reproductive	Fecundity ~ s(sampling_point, bs = 'cs', k = 8)	44	8	0	0.000995	3.79597706678947e-06	96.82971	197.28	0.380444	NS
**Fecundity**	Combined	Reproductive	Fecundity ~ s(sampling_point, bs = 'cs', k = 10) + s(colony, bs = 're') + factor(year)	83	10	0	3.90741	0.015046	179.0561	366.6156	0.435581	NS

Table S6. Temporal variation in estrogen concentrations

Results of (A) Kruskal-Wallis tests and (B) Dunn post-hoc comparisons for estrogen levels by sampling point and year. Sampling points correspond to dates in Table S1. Only statistically significant pairwise differences ($p < 0.05$, Bonferroni-corrected) are shown.

(A) Kruskal-Wallis tests by year

Year	*n*	Chi-squared	df	p-value	Interpretation
**2021**	82	42.92	12	2.33e-05	Significant differences
**2022**	106	53.40	19	4.08e-05	Significant differences
**2023**	93	29.09	10	0.00120	Significant differences

(B) Dunn post-hoc pairwise comparisons

2021:

Sampling Point (From)	Sampling Point (To)	Adjusted p-value	Significance
**1**	9	0.0447	*
**1**	10	0.0212	*
**2**	9	0.0055	**
**2**	10	0.0023	**
**6**	9	0.0272	*
**6**	10	0.0121	*
**7**	10	0.0375	*

2022:

Sampling Point (From)	Sampling Point (To)	Adjusted p-value	Significance
**14**	21	0.0429	*
**14**	23	0.0344	*
**18**	23	0.0476	*

2023:

Sampling Point (From)	Sampling Point (To)	Adjusted p-value	Significance
**34**	42	0.0450	*
**37**	41	0.0263	*

**37**	42	0.0010	**

Table S7. Correlations between steroid hormones and reproductive parameters

Spearman rank correlations for pooled data from 2021-2023. n varies by parameter pair. *p < 0.05; **p < 0.01; ***p < 0.001; NS = not significant. Negative correlations between estrogen and oocyte diameter indicate elevated estrogen during early gametogenesis.

Variable 1	Variable 2	*n*	Spearman's ρ	p-value	Significance	Interpretation
**Estrogen**	Oocyte Diameter	86	-0.321	0.003	**	Inverse correlation: higher estrogen during early gametogenesis when oocytes are smaller
**Estrogen**	Progesterone	240	0.333	<0.001	***	Positive correlation: coordinated hormone variation
**Estrogen**	Fecundity	83	-0.121	0.277	NS	No correlation: estrogen does not regulate oocyte number
**Progesterone**	Oocyte Diameter	83	-0.063	0.572	NS	No correlation with developmental stage
**Progesterone**	Fecundity	80	-0.139	0.220	NS	No correlation: progesterone does not regulate oocyte number

Table S8. GAM results for environmental drivers of estrogen and oocyte diameter

Models tested the influence of photoperiod, UV radiation, water temperature, and PAR on estrogen and oocyte diameter. Colony was included as a random effect in combined-year analyses. k = basis dimension; EDF = effective degrees of freedom; Dev. Expl. = deviance explained; GCV = generalized cross-validation score; AIC = Akaike Information Criterion. *p < 0.05; **p < 0.01; ***p < 0.001.

(A) Estrogen vs Environmental Factors

Year/ Model	Factor	*n*	k	EDF	Dev. Expl. (%)	R^2	GCV	AIC	k-index	p-value	Sig.
**2021 (bi-weekly)**	Photoperiod	82	5	4.21	37.0	0.337	2129.4	862.7	1.13	<0.000001	***
	UV Radiation	82	5	1.63	22.8	0.198	2226.6	862.4	0.94	0.000012	***
	Water Temp	82	5	1.95	18.5	0.157	2360.5	869.0	0.89	0.000343	***
	PAR	82	5	3.58	25.1	0.219	2213.2	872.1	0.99	0.000183	***
**2022 (bi-weekly)**	Photoperiod	72	5	1.72	37.5	0.347	2099.3	753.5	0.90	<0.000001	***
	UV Radiation	72	5	1.64	27.1	0.238	2444.1	763.4	0.86	0.000006	***
	Water Temp	71	5	2.46	31.5	0.284	2350.0	754.0	0.88	0.000005	***
	PAR	72	5	2.44	28.3	0.251	2431.8	767.1	0.83	0.000024	***
**2023 (monthly)**	Photoperiod	93	5	2.32	21.1	0.182	2635.6	997.8	1.09	0.000042	***
	UV Radiation	93	5	2.10	23.7	0.207	2544.3	994.5	1.02	0.000008	***
	Water Temp	92	5	1.04	4.6	0.019	3161.5	998.1	0.91	0.058	NS
	PAR	93	5	4.63	22.7	0.193	2650.8	1015.2	1.06	0.001	**
**Combined (+ year)**	Photoperiod	247	7	5.13	32.5	0.298	2220.0	2606.6	1.10	<0.000001	***
	UV Radiation	247	7	4.01	26.7	0.240	2400.9	2619.9	0.93	<0.000001	***
	Water Temp	245	7	2.84	17.9	0.151	2692.7	2620.3	0.86	<0.000001	***
	PAR	247	7	3.60	13.2	0.104	2838.0	2658.5	0.83	0.000031	***

Multi-Factor Estrogen Model (Combined Years, n=245): 36.3% deviance explained, GCV=2125.9, AIC=2578.7

Smooth Term	EDF	F-statistic	p-value	Interpretation
**s(Photoperiod)**	4.19	8.23	<0.000001	Primary predictor
**s(UV)**	1.41	0.91	0.034	Secondary predictor

**s(Water Temp)**	1.13	1.03	0.014	Secondary predictor
**s(PAR)**	0.01	0.002	0.328	Not significant when controlling for other factors

(B) Oocyte Diameter vs Environmental Factors

Factor	*n*	k	EDF	Dev. Expl. (%)	R^2	GCV	AIC	k-index	p-value	Sig.
**Photoperiod**	86	7	3.01	80.1	0.788	2055.3	887.6	0.74	<0.000001	***
**UV Radiation**	86	7	5.35	70.1	0.682	3178.2	938.5	0.67	<0.000001	***
**Water Temp**	84	7	2.30	72.2	0.705	2782.5	888.4	0.69	<0.000001	***
**PAR**	86	7	5.70	73.7	0.720	2811.6	931.8	0.65	<0.000001	***

Multi-Factor Oocyte Diameter Model (n=84): 86.2% deviance explained, GCV=1442.8, AIC=845.0

Smooth Term	EDF	F-statistic	p-value	Interpretation
**s(Photoperiod)**	2.63	11.37	<0.000001	Primary predictor
**s(UV)**	0.0004	<0.001	0.552	Not significant in multi-factor model
**s(Water Temp)**	2.38	6.40	<0.000001	Secondary predictor
**s(PAR)**	0.76	0.39	0.075	Not significant in multi-factor model

Low k-index values (<0.8) for oocyte diameter models indicate strong environmental signal. All models achieved full convergence (REML with outer Newton optimizer), showed approximately normal Q-Q plots, and homoscedastic residual patterns.

Table S9. Spatial and temporal differences in physiological parameters

Analyses based on eight *Acropora eurystoma* colonies sampled in March and June 2024 (n = 24 per location per month; n = 32 for lipids). Welch t-tests compare regions within months; two-way ANOVA tests effects of location, month, and their interaction. *p < 0.05; **p < 0.01; ***p < 0.001; NS = not significant.

(A) Physiological Parameters

Parameter	Month	Central (Mean ± SE)	Peripheral (Mean ± SE)	Difference (%)	t-test p-value	Significance
**Protein (mg cm⁻²)**	March	0.36 ± 0.02	0.32 ± 0.02	+11.4%	0.280	NS
	June	0.38 ± 0.02	0.32 ± 0.02	+15.6%	0.126	NS
**Estrogen (pg cm⁻²)**	March	138.59 ± 16.57	130.90 ± 15.82	+5.9%	0.762	NS
	June	113.98 ± 11.98	117.69 ± 14.42	-3.1%	0.834	NS
**Lipids (mg cm⁻²)**	March	0.42 ± 0.08	0.48 ± 0.10	-11.3%	0.668	NS
**n=8 per group**	June	0.46 ± 0.11	0.57 ± 0.14	-18.2%	0.502	NS
**Algal density (10^6 cells cm^{-2})**	March	2.29 ± 0.12	1.75 ± 0.08	+31.0%	0.0004	***
	June	1.83 ± 0.10	1.27 ± 0.09	+43.8%	<0.0001	***
**Chlorophyll a (µg cm⁻²)**	March	3.60 ± 0.21	2.27 ± 0.12	+58.8%	<0.0001	***
	June	2.40 ± 0.17	1.33 ± 0.11	+80.9%	<0.0001	***
**Chlorophyll a (pg cell⁻¹)**	March	1.66 ± 0.09	1.32 ± 0.08	+25.7%	0.020	*
	June	1.34 ± 0.08	1.06 ± 0.07	+26.5%	0.018	*

(B) Two-Way ANOVA Results

Parameter	Effect	df	Sum Sq	Mean Sq	F-value	p-value	Sig.
**Protein**	Location	1	0.0456	0.0456	3.502	0.065	.
	Month	1	0.0029	0.0030	0.227	0.635	NS
	Location×Month	1	0.0012	0.0012	0.089	0.766	NS
	Residuals	92	1.1971	0.0130	-	-	-
**Estrogen**	Location	1	96	96	0.017	0.897	NS
	Month	1	8,582	8,582	1.507	0.223	NS
	Location×Month	1	779	779	0.137	0.712	NS
	Residuals	92	523,967	5,695	-	-	-
**Lipids**	Location	1	0.0492	0.0492	0.658	0.424	NS
	Month	1	0.0344	0.0344	0.460	0.503	NS
	Location×Month	1	0.0048	0.0048	0.065	0.801	NS
	Residuals	28	2.0936	0.0748	-	-	-
**Algal density**	Location	1	7.24×10^{12}	7.24×10^{12}	37.260	<0.0001	***
	Month	1	5.26×10^{12}	5.26×10^{12}	27.037	<0.0001	***
	Location×Month	1	1.26×10^{9}	1.26×10^{9}	0.006	0.936	NS
	Residuals	92	1.79×10^{13}	1.94×10^{11}	-	-	-
**Chlorophyll a per area**	Location	1	34.84	34.84	59.173	<0.0001	***
	Month	1	27.49	27.49	46.688	<0.0001	***
	Location×Month	1	0.40	0.40	0.686	0.410	NS
	Residuals	92	54.16	0.59	-	-	-
**Chlorophyll a per cell**	Location	1	2.297	2.297	11.765	0.0009	***
	Month	1	2.057	2.057	10.538	0.002	**
	Location×Month	1	0.021	0.021	0.107	0.744	NS
	Residuals	92	17.960	0.195	-	-	-

(C) Oocyte Prevalence (March 2024, n=48 polyps examined)

Chi-square test: $\chi^2 = 14.49$, df = 1, p = 0.0001

Location	Polyps without oocytes	Polyps with oocytes	Prevalence (%)	Total
**Central**	7	17	70.8%	24
**Peripheral**	21	3	12.5%	24

Interpretation: Central regions had 5.7-fold higher oocyte prevalence than peripheral regions (70.8% vs. 12.5%).

(D) Oocyte Parameters (March 2024, samples with detectable oocytes only)

Parameter	Central (*n*=17)	Peripheral (*n*=3)	t-test p-value	Significance
**Oocyte diameter (µm)**	223.4 ± 5.8	217.7 ± 11.3	0.659	NS
**Fecundity (oocytes/polyp)**	20.2 ± 1.0	21.1 ± 2.3	0.752	NS

Low peripheral sample size (*n*=3) reflects low oocyte prevalence in peripheral regions. Among polyps that did contain oocytes, developmental stage and fecundity were similar between locations.

(E) Temporal Changes Within Locations (March to June)

Parameter	Central Change	Peripheral Change	Both locations significant
**Algal density**	-20.1%	-27.2%	Yes ($p<0.001$)
**Chlorophyll a per area**	-33.3%	-41.4%	Yes ($p<0.001$)
**Chlorophyll a per cell**	-19.4%	-19.9%	Yes ($p=0.002$)
**Protein**	+5.6%	+0%	No ($p=0.635$)
**Estrogen**	-17.8%	-10.1%	No ($p=0.223$)
**Lipids**	+9.5%	+18.8%	No ($p=0.503$)

Both central and peripheral regions showed significant seasonal declines in symbiont density and photosynthetic pigments from March (mid-gametogenesis) to June (pre-spawning), with no significant location×month interaction (all $p>0.4$), indicating parallel seasonal patterns across colony regions.
